# The identification of two M20B family peptidases required for full virulence in *Staphylococcus aureus*

**DOI:** 10.3389/fcimb.2023.1176769

**Published:** 2023-07-19

**Authors:** Nathanial J. Torres, Devon N. Rizzo, Maria A. Reinberg, Mary-Elizabeth Jobson, Brendan C. Totzke, Jessica K. Jackson, Wenqi Yu, Lindsey N. Shaw

**Affiliations:** Department of Molecular Biosciences, University of South Florida, Tampa, FL, United States

**Keywords:** *S. aureus*, peptidases, virulence, pathogenesis, regulation

## Abstract

We have previously demonstrated that deletion of an intracellular leucine aminopeptidase results in attenuated virulence of *S. aureus*. Herein we explore the role of 10 other aminopeptidases in *S. aureus* pathogenesis. Using a human blood survival assay we identified mutations in two enzymes from the M20B family (PepT1 and PepT2) as having markedly decreased survival compared to the parent. We further reveal that *pepT1*, *pepT2* and *pepT1/2* mutant strains are impaired in their ability to resist phagocytosis by, and engender survival within, human macrophages. Using a co-infection model of murine sepsis, we demonstrate impairment of dissemination and survival for both single mutants that is even more pronounced in the double mutant. We show that these enzymes are localized to the cytosol and membrane but are not necessary for peptide-based nutrition, a hallmark of cell-associated aminopeptidases. Furthermore, none of the survival defects appear to be the result of altered virulence factor production. An exploration of their regulation reveals that both are controlled by known regulators of the *S. aureus* virulence process, including Agr, Rot and/or SarA, and that this cascade may be mediated by FarR. Structural modeling of PepT1 reveals it bears all the hallmarks of a tripeptidase, whilst PepT2 differs significantly in its catalytic pocket, suggesting a broader substrate preference. In sum, we have identified two M20B aminopeptidases that are integral to *S. aureus* pathogenesis. The future identification of protein and/or peptide targets for these proteases will be critical to understanding their important virulence impacting functions.

## Introduction

*Staphylococcus aureus* is an opportunistic pathogen widely prevalent amongst the human population, and a leading cause of skin and soft tissue infections, and life-threatening diseases such as endocarditis, osteomyelitis, and necrotizing pneumonia (Turner et al., 2019). The extended pathogenicity of *S. aureus* is largely the result of its repertoire of virulence factors, such as proteases, nucleases, adhesins, hemolysins and specific toxins, all of which allow this bacterium to invade, adhere to, and disseminate throughout host tissues and the bloodstream (Cheung et al., 2021). While there has been extensive research on the contribution of secreted proteases to *S. aureus* pathogenesis (Shaw et al., 2004; Kolar et al., 2013), very little is known about the impact of cell associated proteases on virulence.

Aminopeptidases are a class of exopeptidases that catalyze the cleavage of N-terminal amino acid(s) from proteins and peptides (Sapio and Fricker, 2014). These enzymes are widely distributed throughout nature, and are most commonly localized within the cytoplasm or to membranes, although secreted examples do exist (Taylor, 1993). Typically, aminopeptidases play important roles in growth and metabolism by liberating amino acid constituents from peptides imported into the cell, which can then be used in anabolic pathways (Gonzales and Robert-Baudouy, 1996). These enzymes are also necessary for the catabolism and elimination of abnormal proteins and signal sequences derived from secreted proteins (Goldberg and Dice, 1974; Goldberg and St. John, 1976; Gonzales and Robert-Baudouy, 1996). Furthermore, aminopeptidases can play an integral role in the bioactivation and inactivation of key protein targets. For example, the N-terminus of most bacterial proteins is cleaved by methionine aminopeptidase to alter the function of newly synthesized proteins (Barsun et al., 2007; Abid et al., 2009; Hearn et al., 2009; Naamati et al., 2009; Snelgrove et al., 2010). The importance of such a process is indicated by the essentiality of almost all methionine aminopeptidase enzymes (Auld, 2013).

Of special interest, aminopeptidases have recently been shown to be involved in pathogenesis. In previous work by our group, we revealed that deletion of a *S. aureus* leucine aminopeptidase (LAP/*pepZ*) resulted in attenuated virulence in both systemic and localized models of infection (Carroll et al., 2012; Carroll Ronan et al., 2013). Importantly, LAP marked the first intracellular bacterial aminopeptidase found to be involved in pathogenesis in Gram-positive bacteria. Although the exact molecular mechanisms by which LAP functions is still unknown, it is hypothesized the enzyme is primarily responsible for the bioactivation/inactivation of protein substrates within the cell that are involved in the secretion of virulence factors [Carroll and Shaw, unpublished observation].

Outside of the M17 family of leucine aminopeptidases, few additional bacterial aminopeptidases have been found to play a role in virulence. Two aminopeptidases, a leucine aminopeptidase and aminopeptidase T, were found to be upregulated in the proteomes of virulent strains of *Streptococcus suis*, however their exact roles in pathogenesis are still undetermined (Wang et al., 2011). Additionally, a *pepN* aminopeptidase mutant in *Salmonella typhimurium* resulted in increased bacterial recovery from the spleen, lymph node and thymus during murine infection (Patil et al., 2007). In this work, PepN was found to be important for restricting the number of bacteria during later stages of systemic infection to minimize damage to the host, thus allowing for sustained and successful infection (Patil et al., 2007). Furthermore, a cytoplasmic M29 family aminopeptidase, Lmo1603, in *Listeria monocytogenes* was shown to be important for virulence within a mouse model of infection and was essential for survival and invasion of human epithelial cells and murine macrophages. Interestingly, Lmo1603 was not required for growth on either minimally defined or rich medium (Cheng et al., 2015).

Given our discovery that LAP is required for *S. aureus* disease causation, we set out to explore the role of the remaining, non-essential, uncharacterized aminopeptidase enzymes in *S. aureus* pathogenesis. Herein, we identified two *S. aureus* aminopeptidases, PepT1 and PepT2, that belong to the M20B family and are required for virulence in both *ex vivo* models of human infection and *in vivo* models of murine infection. Notably, these enzymes are not secreted nor found on the bacterial cell surface, and are not necessary for the utilization of free peptides for cellular nutrition and metabolism; which is a typical characteristic of cell-associated aminopeptidases.

## Materials and methods

### Strains, plasmids, primers, and growth conditions

All bacterial strains, plasmids, and primers used in this study are listed in **Tables 1** and **2**. Growth of *S. aureus* and *E. coli* cultures were performed routinely in 100 mL volumes (1:2.5 flask/volume ratio) of tryptic soy broth (TSB) or lysogeny broth (LB), respectively, overnight at 37°C and 250 rpm. When necessary, antibiotics were added at the following concentrations: 100 μg ml^-1^ ampicillin (*E. coli*); chloramphenicol 5 μg ml^-1^, tetracycline 5 μg ml^-1^, erythromycin 5 μg ml^-1^, lincomycin 25 μg ml^-1^, spectinomycin 5 μg ml^-1^ (*S. aureus*). To obtain cultures at specific growth phases, all cultures were first synchronized by using 1mL of the overnight culture to inoculate fresh media, followed by 3 h growth, as described previously (Kolar et al., 2011). The optical density of the synchronized culture was then standardized to a OD_600 =_ 0.05, unless otherwise stated, and allowed to grow until the desired growth phase was reached.

**Table 1:** Strains and Plasmids.

Strain or Plasmid	Genotype/properties^a^	Reference or source
E. coli		
DH5α	Cloning strain	(Salisbury et al., 1972)
S. aureus		
USA300 HOU	USA300-HOU MRSA cured of pUSA300-HOU-MRSA	(Kolar et al., 2011)
RN4220	Restriction-deficient strain	Lab stocks
NE 921	USA300 JE2 *pepA1*::Tn::Erm, *pepA1^-^*	(Fey et al., 2013)
NE 1264	USA300 JE2 *pepA2*::Tn::Erm, *pepA2^-^*	(Fey et al., 2013)
NE 1306	USA300 JE2 *pepA3*::Tn::Erm, *pepA3^-^*	(Fey et al., 2013)
NE 1169	USA300 JE2 *pepF1*::Tn::Erm, *pepF1^-^*	(Fey et al., 2013)
NE 195	USA300 JE2 *pepF2*::Tn::Erm, *pepF2^-^*	(Fey et al., 2013)
NE 483	USA300 JE2 *pepT1*::Tn::Erm, *pepT1^-^*	(Fey et al., 2013)
NE 519	USA300 JE2 *pepT2*::Tn::Erm, *pepT2^-^*	(Fey et al., 2013)
NE 1482	USA300 JE2 *pepP1*::Tn::Erm, *pepP1^-^*	(Fey et al., 2013)
NE 306	USA300 JE2 *pepP2*::Tn::Erm, *pepP2^-^*	(Fey et al., 2013)
NE 1898	USA300 JE2 *pepV*::Tn::Erm, *pepV^-^*	(Fey et al., 2013)
NE 444	USA300 JE2 *pepS*::Tn::Erm, *pepS^-^*	(Fey et al., 2013)
DNM1868	USA300 HOU *pepT1*::Tn::Erm, *pepT1^-^*	This study
DNM1867	USA300 HOU *pepT2*::Tn::Erm, *pepT2^-^*	This study
DNM2189	USA300 HOU *pepT1*::Tn::Spc/*pepT2*::Tn::Erm, *pepT1*/*pepT2* double mutant	This study
DNM1994	USA300 HOU *pepT1*::Tn::Erm/pMK4::*pepT1*-His_6_, *pepT1* ^+^	This study
DNM1995	USA300 HOU *pepT2*::Tn::Erm/pMK4::*pepT2*-His_6_, *pepT2* ^+^	This study
NJT3436	USA300 HOU pXen::P*_pepT1_*-*luxABCDE*	This study
NJT3437	USA300 HOU pXen::P*_pepT2_*-*luxABCDE*	This study
Plasmids		
pMK4	Gram-positive shuttle vector	(Sullivan et al., 1984)
pSpc	Plasmid for spectinomycin resistance cassette switch *via* allelic exchange and homologous recombination	(Bae et al., 2008)
pXen-1	Promoterless *luxABCDE* shuttle vector	(Francis et al., 2000)
pDNM01	pMK4::*pepT1*-His_6_	This study
pDNM02	pMK4::*pepT2*-His_6_	This study
pNJT01	pXen::P*_pepT1_*-*luxABCDE*	This study
pNJT02	pXen::P*_pepT2_*-*luxABCDE*	This study

a Erm, erythromycin; Spc, spectinomycin. Tn indicates transposon insertion.

**Table 2:** Primer Sequences.

Primer	Primer Sequence^a^	Description^b^
OL761	GACCATGCGAG GATGATGC	R pAZ106
OL2393	TCGTATGTTGTGTGGAATTG	F pMK4 screening
OL2394	GTGCTGCAAGGCGATTAAG	R pMK4 screening
OL5416	ATCAGAGCAGATTGTACTGAG	F pXen screening
OL5417	ACTCCTCAGAGATGCGAC	R pXen screening
OL3112	AAAGAATTCTTAGAAGTTACACTGATTTCAGG	F pMK4 *pepT1*
OL3113	TTTCCCGGGTTA**GTGGTGGTGGTGGTGGTG**ATTTTCAGCG	R pMK4 *pepT1*-His_6_
OL3110	AAAGAATTCTATAAAACTTTTAACTTGAGACG	F pMK4 *pepT2*
OL3111	TTTCCCGGGTTA**GTGGTGGTGGTGGTGGTG**TTTCGAATGTCGCGC	R pMK4 *pepT2-*His_6_
OL6690	ATGGAATTCCGGTAGATTTAGAGTTTGGAAAAGTTGGCG	F pXen P*_pepT1_*-*luxABCDE*
OL6691	ATGGGATCCTAGTATAAGCAAAATCAGCATTGAAGCGG	R pXen P*_pepT1_*-*luxABCDE*
OL6692	ATGGAATTCGATTTTGTATAGGTAG	F pXen P*_pepT2_*-*luxABCDE*
OL6693	ATGGGATCCCAACAGTATCCATATGGC	R pXen P*_pepT2_*-*luxABCDE*

a Restriction sites are underlined and His_6_ tags are bolded.

b F, forward; R, reverse.

### Mutant strain construction

Both the *pepT1* (USA300 JE2 *SAUSA300_0727*) and *pepT2* (USA300 JE2 *SAUSA300_1460*) mutants were obtained from the Nebraska Mutant Transposon Library (Fey et al., 2013). These were used to make phi11 phage lysates that were transduced into the *S. aureus* USA300 HOU background and confirmed by PCR using primer pairs OL3112/OL3113 and OL3110/OL3111, respectively. The *pepT1/pepT2* double mutant was created by first exchanging the erythromycin resistant cassette in the *pepT1* mutant using pSPC, a genetic exchange plasmid designed to incorporate alternate antibiotic resistance markers while subsequently removing the erythromycin cassette found in the *bursa aurealis* transposon (Bae et al., 2008; Bose et al., 2013; Bose, 2014). The *pepT1* spectinomycin mutant was confirmed *via* PCR and used to make a phi11 phage lysate that was subsequently transduced into the *pepT2* erythromycin marked mutant.

### Construction of *pepT1* and *pepT2* complementing strains

Both the *pepT1* and *pepT2* complementing strains were created as previously described (Miller et al., 2012). Briefly, primer pairs OL3112/OL3113 and OL3110/OL3111 were used to PCR amplify both *pepT1* and *pepT2*, respectively, along with their native promoters. Primers OL3113 and OL3111 also added a hexahistidine (His_6_) tag at the C-terminal end of each aminopeptidase. PCR fragments were then cloned into shuttle vector pMK4 and transformed into *E. coli* DH5α. Both constructs were confirmed by DNA sequencing and subsequently electroporated into *S. aureus* RN4220 (Kreiswirth et al., 1983). Upon PCR confirmation using plasmid specific primers, phi11 phage lysates were created and used to transduce the respective single mutant strains. The final complement constructs were verified by PCR and sequencing.

### Construction of reporter fusion strains

*pepT1- and pepT2-*luciferase fusion transcriptional reporter strains were constructed as previously described (Weiss et al., 2022). Briefly, primer pairs OL6690/OL6691 and OL6692/OL6693 were used to PCR amplify the promoters of *pepT1* and *pepT2*, respectively. Each PCR fragment was cloned into pXen-1 (Francis et al., 2000), which contains a promoterless *luxABCDE* operon, and transformed into *E. coli* DH5α. Both constructs were confirmed by DNA sequencing and electroporated into *S. aureus* RN4220. Upon PCR confirmation using plasmid specific primers OL5416 and OL5417, each construct was used to create phi11 phage lysates, before being mass transduced into mutants of every known transcriptional regulator within the NTML (Fey et al., 2013), alongside the wild-type strain, as previously described (Weiss et al., 2022).

### Reporter-fusion assays

Luciferase reporter assays using the luciferase reporter fusion strains were performed as previously described (Weiss et al., 2022). Briefly, 2 μL of overnight culture for wild-type or regulator mutant strains bearing pNJT01 or pNJT02 were used to inoculate 198 μL of fresh TSB and allowed to grow for 3 h. Cultures were then standardized to an OD_600_ of 0.05 in fresh TSB inside black walled 96-well plates and allowed to grow for 8 h. Luminescence was measured every 15 mins using a Cytation 5 imaging plate reader (BioTek) as previously described (Weiss et al., 2022). To determine significant alterations in promoter activity, area under the curve was initially calculated between 2-5 h for each strain using a 1.5-fold cutoff relative to the wild-type (**Supplemental Table 1**). Two-way ANOVA analysis, with Sidaks multiple comparisons test, was then used to assess statistical significance for RLU production in mutants by comparison to the wild-type strain. Results are presented as the average of 3 independent experiments.

### Survival in whole human blood

Blood survival assays were performed as previously described (Weiss et al., 2014). Briefly, synchronized cultures for strains were grown to exponential phase, and subsequently washed three times in PBS. Cultures were then diluted to an OD_600_ of 0.05 and added to 1 mL of whole human blood (BioIVT). The initial inoculum for each strain was determined at this time by plating serial dilutions onto TSA. Each sample was then incubated for 4 h at 37°C, with shaking, and the CFU/mL of each sample was determined by serial dilution and plating on TSA. Data is presented as percent survival for each strain in comparison to the initial inocula. One-way ANOVA analysis, with Dunnett’s multiple comparisons test, was used to assess statistical significance in comparison to the wild-type strain. Results are the average of three biological replicates.

### THP-1 cell line infections

Infections were carried out with THP-1 macrophage-like cells as previously described (Carroll et al., 2014). Briefly, THP-1 human monocytes were grown at 37°C, with 5% CO_2_, in RPMI with L-glutamine and 10% fetal bovine serum. Monocytes were then treated with 80 nM PMA (phorbol 12-myristate 13 acetate) to differentiate them into macrophages. After 48 h, the THP-1 cells were seeded into a 96-well microtiter plate at a density of 1x10^6^ cells/well, and subsequently infected with 1x10^6^ CFU/mL of the wild-type, *pepT1*, *pepT2*, and *pepT1/T2* mutant strains. Plates were then centrifuged for 10 mins at 1000 x g, and phagocytosis was allowed to progress for 1 h at 37°C with 5% CO_2_. Following phagocytosis, each sample was washed three times with PBS, and treated with 30 μg mL^-1^ gentamycin for 1 h at 37°C with 5% CO_2_ to kill any remaining extracellular bacteria. To determine the number of bacteria phagocytized, THP-1 macrophages were washed three times in PBS and treated with 0.5% Triton X 100 to lyse macrophages. Cellular lysates were serially diluted and plated onto TSA. One-way ANOVA analysis, with Dunnett’s multiple comparisons test, was used to assess statistical significance in comparison to the wild-type strain. Results are the average of three biological replicates.

To determine intracellular survival of the wild-type, *pepT1*, *pepT2*, and *pepT1/T2* mutant strains after 24h, all the steps above, up to treating each sample with 30 μg mL^-1^ gentamycin for 1h, were followed. However, after the addition of gentamycin, all media was removed from each sample and replaced with media containing 5 μg mL^-1^ of gentamycin. Samples were then incubated at 37°C with 5% CO_2_ for an additional 24h, before treatment with 0.5% Triton X 100 to lyse the THP-1 cells. Cellular lysates were then serially diluted and plated onto TSA. Data is presented as percent phagocytosed or percent survival for each strain in comparison to the initial inoculum. One-way ANOVA analysis, with Dunnett’s multiple comparisons test, was used to assess statistical significance in comparison to the wild-type strain. Results are the average of three biological replicates.

### Co-infection models of systemic infection

Co-infection models of systemic infection were carried out as previously described (Weiss et al., 2014). Three sets of 10 mice were separately co-infected at the same time with the wild-type strain and a *pepT* mutant. The inoculating dose was a combined 5 x 10^7^ CFU/mL of the wild-type and *pepT1*, *pepT2*, or *pepT1/T2* mutant strains in a 1:1 ratio. The infection was allowed to progress for 7 days, after which time, all mice were euthanized. The kidneys, brain, heart, liver, lungs, and spleen were harvested and homogenized in 3mL PBS. Each sample was then serially diluted and plated, in duplicate, on TSA and TSA containing erythromycin (to select for mutant strains only) and allowed to incubate overnight at 37°C. After this time the CFU/organ was determined for wild-type and mutant strains. The competitive index was calculated by: (output mutant CFU/output WT CFU)/(input mutant CFU/input WT CFU). A CI value < 1 indicates a fitness defect for the mutant strain in comparison to the wild-type. A Wilcoxon signed rank test was used to determine statistical significance.

### Protein extraction and Western blot analysis

Cytoplasmic, membrane, and secreted protein fractions were extracted from 100mL of synchronized cultures after 15 h, as described previously (Rivera et al., 2012). Immediately following growth, all strains were adjusted to equal optical densities. Then, cultures were centrifuged for 10 mins at 4150 rpm after which time the supernatant was collected (secreted protein fraction). The remaining cell pellets were then resuspended in 1 mL of lysis buffer (500 μg/mL lysostaphin, 100 U DNase I, 25 U Rnase I, 2X Pierce Protease inhibitor cocktail (Thermofisher)) and incubated for 30 mins at 37°C. Samples were then subject to 5 rounds of bead beating for 45 s each, with 1 min rest periods in-between. Next, samples were centrifuged at 17,000 x g for 10 mins, at which time the supernatant was collected (intracellular protein fraction). The cell pellet (cell wall protein fraction) was then thrice washed with PBS and resuspended in 1 mL SDS lysis buffer (50 mM TEAB, 5% (w/v) SDS).

The surface exposed cellular fraction was obtained as previously described (Zheng et al., 2021). Briefly, cell pellets were obtained from 15 h cultures as described above. Each sample was then washed with PBS and subsequently incubated with 1X Pierce Protease inhibitor cocktail in PBS for 10 mins at 37°C. Next, SDS was added to each sample at a final concentration of 1% (w/v) and incubated for an additional 30 mins at room temperature. All samples were then centrifuged at 13,000 rpm for 2 min, after which the supernatant was collected (surface-exposed fraction).

Western blots were performed as previously described (Carroll et al., 2014). Briefly, cytoplasmic, membrane, and secreted protein fractions were loaded in equal amounts and run on 12% SDS-PAGE before being transferred onto a PVDF membrane, as previously described (Marroquin et al., 2019). Immunoblotting was performed using: an anti-His_6_ rabbit polyclonal antibody (Thermofisher), an anti-RpoB mouse monoclonal antibody (Santa Cruz Biotechnology), an anti-SrtA rabbit polyclonal antibody, and an anti-Hla rabbit polyclonal antibody (IBT Bioservices). The His_6_ tagged proteins, SrtA, and Hla were detected using a secondary HRP conjugated anti-rabbit antibody and RpoB was detected using a secondary HRP conjugated anti-mouse antibody. Protein bands were visualized on x-ray film.

### Gelatin zymography

Secreted protease activity was assessed by gelatin zymography as previously described (Marroquin et al., 2019). Briefly, strains were grown for 15 h, adjusted to equal optical densities and pelleted. Next, 2 mL of supernatant was loaded into an Amicon Ultra 3K centrifugal filter for 60 min (4,000 X g). After centrifugation, the concentrated filtrate was recovered from collection tubes after inverting the filter devices and spinning again for 2 min (1,000 X g). An equal volume of Laemmeli buffer was added to the concentrated filtrate and incubated for 30 minutes (37°C). Next, aliquots of each sample were electrophoresed on 12% SDS-PAGE gels containing 0.1% gelatin as a substrate for proteolysis. Gels were then washed twice using 2.5% Triton X-100 at room temperature and then thoroughly rinsed with distilled water (dH_2_O). Developing buffer (0.2 M Tris, 5 mM CaCl_2_, 1 mM dithiothreitol [DTT], pH 7.6) was added to gels and incubated statically for 15 h at 37°C. After incubation, gels were washed with dH_2_O and stained using 0.1% amido black for 1 h. After staining, the gels were first destained with Destain 1 (30% methanol, 10% acetic acid) for 20 minutes, replaced with Destain 2 (10% acetic acid) for 30 minutes, and lastly replaced with Destain 3 (1% acetic acid) for storage. Results are representative of three biological replicates.

### Hemolysis assay

Hemolytic activity was assessed as previously described (Marroquin et al., 2019). Briefly, all strains were grown, as described above, and standardized to an OD_600_ of 0.05. Cultures were then incubated for 6 h (37°C), after which time culture supernatants were collected following centrifugation, and mixed (1:1) with hemolysis buffer (0.145 M NaCl, 20 mM CaCl_2_), followed by the addition of 25 μL sheep blood (Hemostat). Samples were then incubated for 40 min (37°C) on a rotator followed by centrifugation at 5.5 x g for 1 min. Supernatants were loaded into 96-well plates and hemolysis was determined by measuring the OD_543_ using a Cytation 5 imaging plate reader (BioTek). One-way ANOVA analysis, with Dunnett’s multiple comparisons test, was used to assess statistical significance in comparison to the wild-type. Results are the average of three biological replicates.

### Protein homology modeling

Homology models for PepT1 and PepT2 were made as previously described (Morellet et al., 2022). Briefly, the crystalized protein structures of PepT from *Bacillus cereus* (PDB ID: 3gb0), PepT from *Salmonella enterica ser. Typhimurium* (PDB ID: 1fno), and PepT2 from *S. aureus* (PDB ID: 3rza) were obtained from the RCSB Protein Data Bank (https://www.rcsb.org/). The predicted structure of PepT1 (UniProt ID: Q2FIP8) was obtained from Alphafold v2 (https://alphafold.ebi.ac.uk/). Structural alignments were generated using PyMOL v2.5.4 (Schrödinger).

## Results

### PepT1 and PepT2 are required for survival in human blood

**Figure 1:**
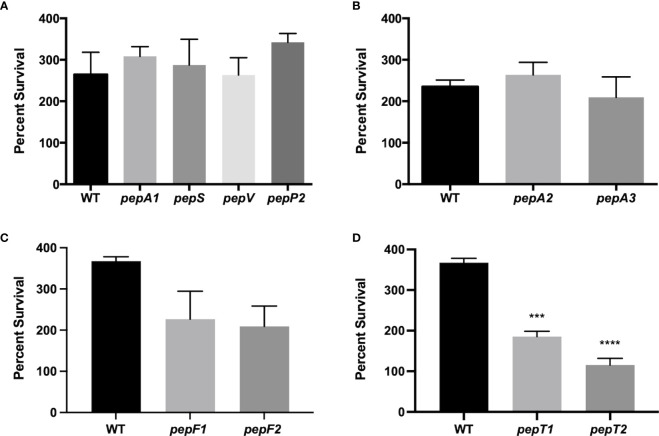
Survival of *S. aureus* aminopeptidase mutants in whole human blood. **(A-D)** Exponentially growing wild-type and aminopeptidase mutant strains were separately inoculated, in triplicate, into whole human blood. Samples were taken after 4 h and the CFU/mL for each was determined. Percent survival was then calculated by comparing the inoculating CFU/mL for each sample to that after 4 h of growth in blood. One-way ANOVA analysis, with Dunnett’s multiple comparisons test, was used to assess statistical significance in comparison to the wild-type, (***, *p* < 0.005; ****, *p* < 0.0005). All data is derived from three independent biological replicates. Error bars are shown ± SEM.

**Figure 2:**
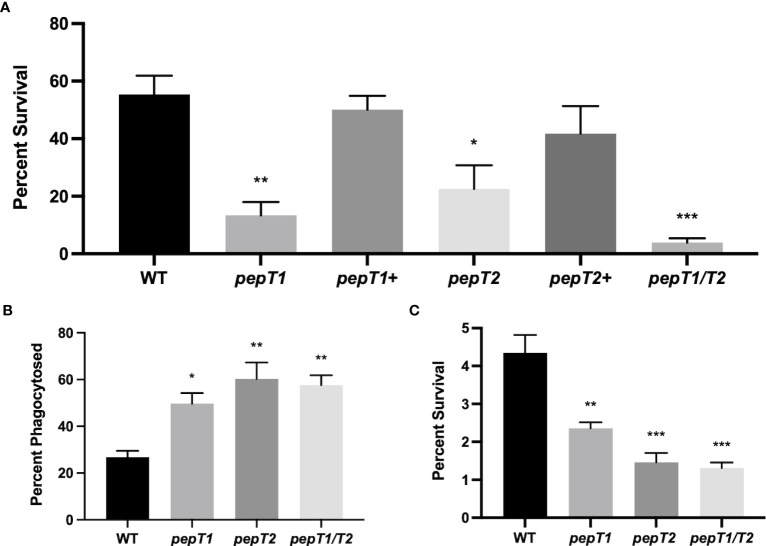
PepT1 and PepT2 are required for *S. aureus e*ngagement with the human immune system. **(A)** As in **Figure 1** using the wild-type, *pepT1, pepT2, pepT1/pepT2* double mutant, as well as *pepT1^+^* and *pepT2^+^* complement strains. **(B)** THP-1 human macrophage-like cells were separately infected with wild-type, *pepT1*, *pepT2*, and *pepT1/T2* double mutant strains. Phagocytosis was allowed to proceed for 1 h Data is presented as the percentage of cells phagocytized (CFU/mL of bacteria phagocytosed/the initial inoculum). **(C)** As in B but intracellular survival was measured after 24h Data is presented as percentage intracellular survival (CFU/mL of bacteria that survived within THP-1 cells after 24 h/CFU/mL of bacteria phagocytosed from 2B). One-way ANOVA analysis, with Dunnett’s multiple comparisons test, was used to assess statistical significance in comparison to the wild-type, (*, *p* < 0.05; **, *p* < 0.005; ***, *p* < 0.0005). All data is derived from three independent biological replicates. Error bars are shown ± SEM.

Previous work by our group characterized the cytoplasmic leucine aminopeptidase LAP/*pepZ* as being necessary for *S. aureus* virulence (Carroll et al., 2012; Carroll Ronan et al., 2013). Given the importance of LAP in *S. aureus* disease causation, we set out to explore whether additional aminopeptidases were important for virulence using a whole human blood survival assay. In so doing, we noted that six of the ten aminopeptidase mutants tested showed no notable differences in survival compared to the wild-type strain (**Figures 1A, B**). Interestingly, however, the M3B (*pepF*) and M20B (*pepT*) family enzymes all had impaired survival compared to the wild-type. Specifically, a *pepF1* mutant demonstrated a 1.6-fold decrease in survival, whilst a *pepF2* mutant demonstrated a 1.7-fold decrease (although neither were found to be statistically significant) (**Figure 1C**). More striking was the M20B enzyme data, (**Figure 1D**), with the *pepT1* and *pepT2* mutants demonstrating a 2-fold and 3.1-fold decrease in viability, respectively. When these studies were repeated using complementing strains for the *pepT* mutants, alongside a newly constructed *pepT1*/*pepT2* double mutant, we again saw major differences in survival for the gene-inactivated strains (**Figure 2A**), which was reversed upon *in trans* complementation. Of more importance, however, was the finding that the double mutant displayed a 14-fold decrease in survival compared to the parental strain. It is noted that the survival values for the single mutants do differ between **Figures 1** and **2**, however these assays were performed on different days with different blood samples; thus variability is to be expected. Regardless, both assays show the same striking defect in survivability for *S. aureus* strains lacking one or both PepT enzymes.

To determine if these findings were simply the result of a defect in growth, we tested the ability of the *S. aureus* aminopeptidase mutants to grow in both nutrient rich media (TSB) and peptide rich media (10% skim milk). The rationale for this latter approach is that in other organisms, such as *Lactobacillus* species (Makarova et al., 2006), aminopeptidases N-terminally cleave imported peptides, releasing free amino acids that can be utilized in metabolic pathways (Sapio and Fricker, 2014). Upon analysis, we observed no difference in growth for any of the strains, as compared to the wild-type, in either condition (**Supplemental Figure S1**). Interestingly, this mirrors data derived from our study of the Leucine Aminopeptidase (LAP) of *S. aureus*, where virulence defects were observed that are not related to growth (Carroll Ronan et al., 2013).

### Investigating the role of PepT1 and PepT2 during leukocyte engagement

To explore the blood survival defect further, we next sought to investigate the role of these enzymes during interaction within host immune cells. As such, immortalized THP-1 human macrophage-like cells were separately infected with 10^6^ CFU/mL of the wild-type, *pepT1*, *pepT2*, and *pepT1/T2* mutant strains, and phagocytosis was allowed to progress for 1h. When survival was determined we noted that 26.7% of the wild-type cells were phagocytosed (**Figure 2B**), whereas ~50% of *pepT1* mutant cells (1.8-fold increase compared to WT) and 60% (2.2-fold increase compared to WT) of *pepT2* mutant cells were phagocytized. Interestingly, the *pepT1/T2* double mutant also displayed enhanced phagocytosis in comparison to the parent strain (60% cells phagocytized, 2.2-fold increase), that mirrored that of the *pepT2* mutant strain. These results indicate that both PepT1 and PepT2 are required to successfully evade phagocytosis. It is important to note that the selecting antibiotics for our complementation constructs are unable to penetrate host immune cells. Moreover, it has shown that without such selection, the majority of complementing plasmids are lost from *S. aureus* cells during leukocyte engagement (Krute et al., 2016). Thus, we were unable to perform complementation with these assays.

To assess the ability of the mutants to survive inside host immune cells, phagocytosis assays were performed again in an identical manner; however, the assay was allowed to proceed for 24 hours before THP-1 cells were lysed. When the percent survival of the wild-type was studied after this time, we determined that 4.3% of the total cells phagocytized survived (**Figure 2C**). By contrast, only 2.4% of *pepT1* mutant cells phagocytized were found to have survived (a 1.8-fold decrease as compared to the wild-type), whilst 1.5% of total *pepT2* mutant cells phagocytized were recovered (a 3-fold decrease as compared to wild-type). Of note, 1.3% of the *pepT1/T2* mutant bacteria survived after 24 h, displaying a 3.3-fold decrease as compared to wild-type.

### Impaired survival of *pepT1* and *pepT2* mutant strains during *ex vivo* infection is not mediated by impaired synthesis of key virulence factors

**Figure 3:**
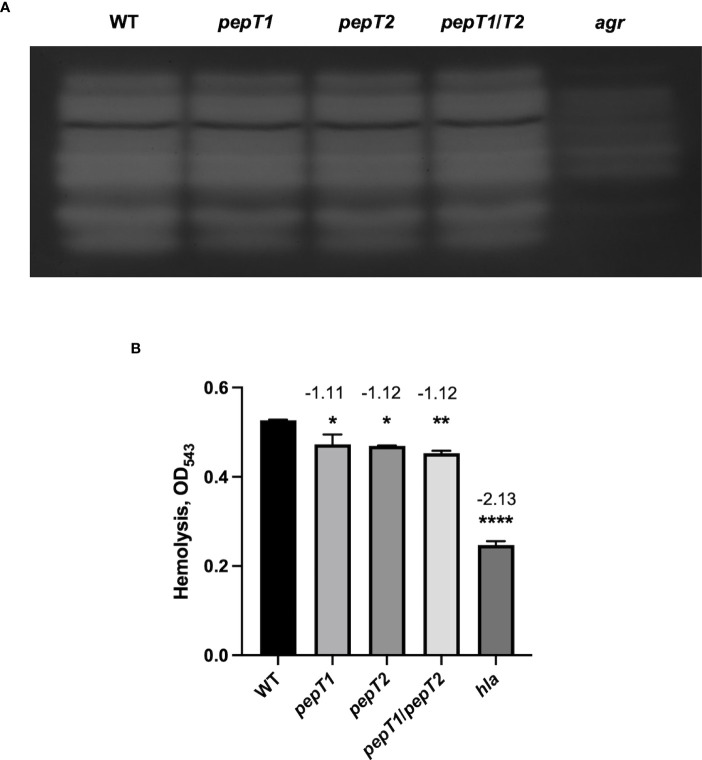
Inactivation of the PepT Enzymes Does Not Impair Proteolytic or Hemolytic Activity. **(A)** Protease activity from 15 h culture supernatants was visualized using gelatin zymography for the wild-type strain and *pepT1*, *pepT2*, *pepT1*/*T2*, and *agr* mutants. All strains were adjusted to equal optical densities prior to analysis. **(B)** Hemolytic activity was measured *via* lysis of erythrocytes in sheep blood. One-way ANOVA analysis, with Dunnett’s multiple comparisons test, was used to assess statistical significance in comparison to the wild-type strain, (*, *p* < 0.05; **, *p* < 0.005; ****, *p* < 0.0001). All data is derived from three independent biological replicates. Error bars are shown ± SEM.

To determine if the virulence related phenotypes seen for the *pepT* mutants were mediated by altered production of virulence factors, we assessed the proteolytic and hemolytic capacity of the *pepT1, pepT2*, and *pepT1/pepT2* mutant strains in comparison to the wild-type and negative control strains (*agr* and *hla* mutants). Using gelatin zymography, we found no difference in proteolytic activity for any of our mutant strains when compared to the parent (**Figure 3A**). The same could not be said for the *agr* mutant. When determining alterations in hemolysis for our strains, we actually observed statistically significant differences for all of our mutant strains (**Figure 3B**). With that said, however, the *pepT* mutant strains had fold changes of only -1.11 and -1.12. Thus, although statistical significance was achieved, there is clearly no biological significance to such limited changes. Indeed, when we tested our *hla* mutant in these assays, it displayed a -2.13 fold change. Thus, our data suggests that these M20B family enzymes do not influence virulence by controlling production or activity of key pathogenic factors.

### Determining the localization and timing of PepT enzyme production during growth in *S. aureus*

**Figure 4:**
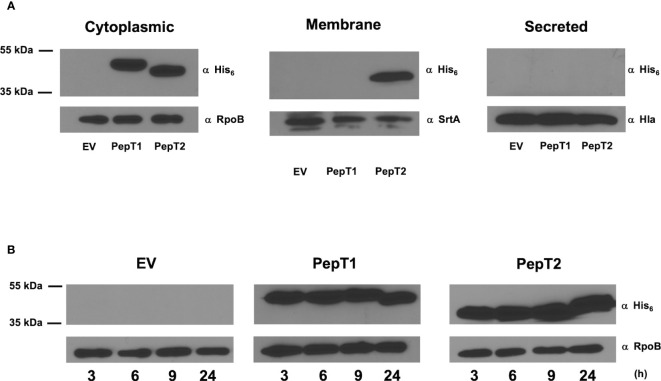
Determining the localization and timing of PepT enzyme production during growth in *S. aureus*. Immunoblots using an anti-His antibody were used to quantify the localization and abundance of both PepT1 and PepT2. **(A)** Immunoblots of cytoplasmic, membrane, and secreted protein fractions collected after 15h growth. The strains used contained plasmid encoded His_6_ tagged PepT1 and PepT2 inside the *pepT1* and *pepT2* mutant backgrounds, respectively. Immediately following growth, all strains were adjusted to equal optical densities prior to cell fractionation. Antibodies specific to the RNA polymerase β subunit (RpoB), sortase A (SrtA), and α-hemolysin (Hla) were used as cell fractionation controls for the cytoplasm, membrane, and secreted protein fractions, respectively. Wild-type USA300 HOU harboring an empty pMK4 vector (EV) was used as a control. **(B)** As in A, but samples were collected at 3, 6, 9, and 24 h from cytoplasmic fractions. All strains were adjusted to equal optical densities prior to analysis.

**Figure 5:**
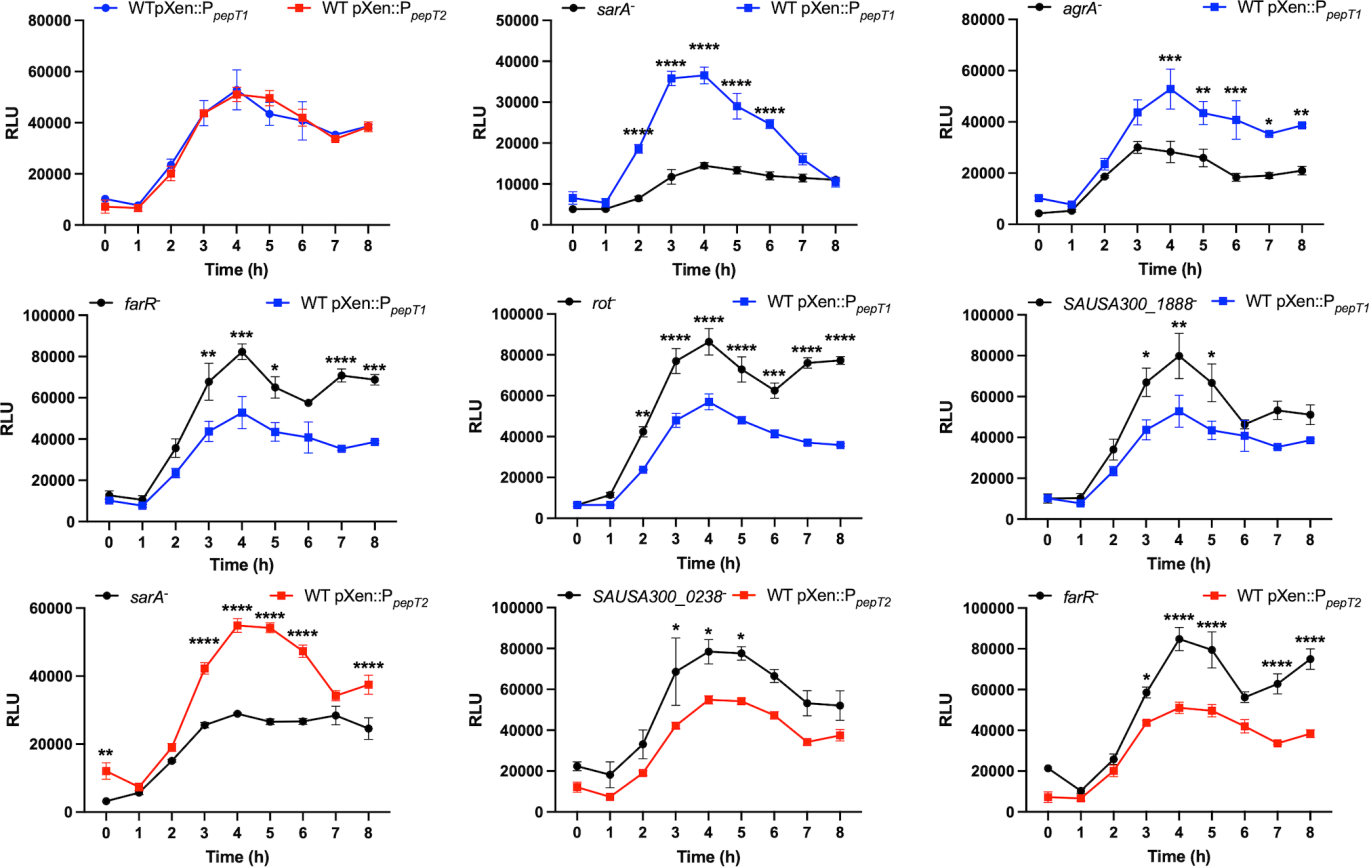
Exploration of *pepT1* and *pepT2* expression and regulation. The first panel shows wild-type strains harboring either a *pepT1*- or *pepT2*-luciferase transcriptional reporter fusion. The following panels show the *pepT1* (blue) or *pepT2* (red) fusions in the wild-type strain or a mutant of the regulator noted. All cultures were standardized to an OD_600 =_ 0.05 and grown for 8h. Luminescence was measured every hour. Two-way ANOVA analysis, with Sidaks multiple comparisons test, was used to assess statistical significance in comparison to the wild-type strains harboring either the *pepT1*- or *pepT2*-luciferase transcriptional reporter fusion, (*, *p* < 0.05; **, *p* < 0.005; ***, *p* < 0.001; ****, *p* < 0.0001) Data is presented as the average of 3 independent experiments with error bars shown as ± SEM.

Despite no evidence of a signal sequence for either PepT1 or PepT2 (data not shown), we next explored their cellular localization. This is of importance, because if these enzymes were found extracellularly, it might then explain their apparent role in virulence (via proteolysis of host factors). As such, C-terminally 6-histidine tagged variants of each enzyme were generated and expressed in their respective mutant strains. Cytoplasmic, cell envelope, and secreted protein fractions were then collected from these strains after 15h of growth, and subjected to Western blot analysis using an anti-His_6_ antibody. As controls, antibodies specific to the RNA polymerase β subunit (RpoB, cytosol), sortase A (SrtA, membrane), and α-hemolysin (Hla, secretome) were also used. Upon analysis, we found that both PepT enzymes were located in the intracellular environment with no evidence of either enzyme seen in the secreted fractions (**Figure 4A**). Interestingly, PepT2 was also found within the cell envelope fraction, which was not a result of contamination because no signal was achieved in this fraction with the RpoB antibody. To determine if PepT2 solely localizes within the membrane and not on the cell surface, we next separated the surface exposed cellular fraction (cell wall) from the cytoplasmic membrane. In so doing, we found no signal for either PepT enzyme (**Supplemental Figure S2**). Thus, it would appear that PepT1 exclusively localizes to the cytosol, whilst the majority of PepT2 is also found in the cytosol, although significant amounts are also present in the membrane. To determine if PepT1 and PepT2 protein levels were altered throughout the *S. aureus* growth cycle, we performed Western blot analysis again, with samples collected at various time intervals. Here, we note that both PepT1 and PepT2 are present at equivalent levels during each time point tested (**Figure 4B**). In order to ascertain if this was from constitutive expression, or stable accumulation, we next created *pepT1* and *pepT2*-luciferase reporter fusion strains. These were grown in TSB, and samples were taken every hour for 8h. Here, we noted that both *pepT1* and *pepT2* have equivalent expression patterns, with maximal transcription occurring during exponential growth (2-5h), followed by a steady decline over time (**Figure 5**). These results suggest that PepT1 and PepT2 are produced in high abundance early in the *S. aureus* lifecycle, remain stably present over time and are not readily degraded in the cell during growth.

### *pepT1* and *pepT2* expression is controlled by known regulators of virulence

Having established that *pepT1* and *pepT2* are highly transcribed during log phase, we next sought to determine how their transcription was controlled within the cell. Thus, our luciferase reporter fusions for both *pepT1* and *pepT2* were transduced into mutant strains representative of all known *S. aureus* transcriptional regulators from the Nebraska Transposon Mutant Library (Fey et al., 2013). As peak promoter activity for both aminopeptidases is between 2 to 5 hours we used this timeframe to assess alterations in promoter activity in the regulator mutants. This was done by initially calculating the area under the curve for each strain (using a 1.5-fold cutoff), in comparison to wild-type, using the method described by Weiss and coworkers (Weiss et al., 2022). Our analysis (**Figure 5**) revealed that both *pepT1* and *pepT2* are repressed by FarR, a regulator of fatty acid resistance, and activated by the global virulence factor regulator SarA. However, *pepT1* alone was repressed by SAUSA300_1888, a TrpR homolog, and Rot, a *sarA* homolog and global repressor of toxins and proteases. Furthermore, *pepT1* was activated by the major global regulator AgrA. While none of these three regulators affected *pepT2* expression, SAUSA300_0238, a BglG transcriptional anti-terminator, repressed *pepT2* promoter activity. Collectively, our analysis reveals that both *pepT1* and *pepT2* are differentially regulated, which may suggest that the cell requires these enzymes during different environmental conditions, and that they could act independently of each other.

### PepT1 and PepT2 are required for survival during systemic infection

**Figure 6:**
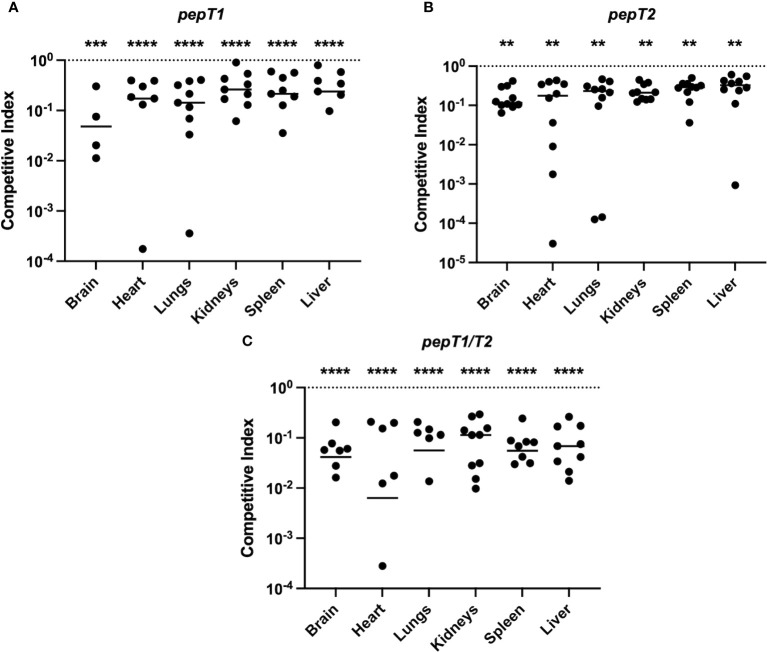
The *S. aureus* PepT enzymes are required for bacterial fitness within a murine model of septic infection. Three sets of 10 mice were separately co-infected at the same time with the wild-type strain and a *pepT* mutant. The inoculating dose was a combined 5 x 10^7^ CFU/mL of the wild-type and **(A)** *pepT1*, **(B)** *pepT2*, or **(C)** *pepT1/T2* mutant strains in a 1:1 ratio. The infection was allowed to progress for 7 days and the mice were subsequently euthanized. Bacterial burdens (CFU/organ) where then quantified from the brain, heart, lungs, kidneys, spleen, and liver. Data is presented as a competitive index **(CI)** with each filled circle representing the CI for one mouse, with bars representing the median CI for each organ. The competitive index was calculated by: (output mutant CFU/output WT CFU)/(input mutant CFU/input WT CFU). The horizontal dotted line represents a CI value of 1.0, which indicates equal survivability. A Wilcoxon signed rank test was used to determine statistical significance from a value of 1, (**, *p* < 0.005; ***, *p* < 0.001; ****, *p* < 0.0001). One mouse from the *pepT1* study died during the 7 day infection period and was omitted from analysis. For organs where 10 symbols are not shown (9 in the case of A), this is because either no colonies were detected for both the mutant and wild-type strains, or no bacterial loads were detected for the mutant strain (and thus cannot be plotted on a logarithmic scale).

Given the significance of both PepT1 and PepT2 in *ex vivo* models of human infection, and that they are regulated by global virulence regulators, we next set out to assess their role during infection using a murine model of sepsis and dissemination. Accordingly, three sets of 10 mice were separately co-infected intravenously with 5 x 10^7^ CFU/mL of wild-type and either the *pepT1*, *pepT2* or *pepT1*/*pepT2* double mutant strains in a 1:1 ratio. The co-infections were allowed to progress for 7 days before the mice were euthanized and organs harvested. One mouse from the *pepT1* study died during the 7 day infection period and was omitted from analysis, however all mice in the *pepT2* and *pepT1*/*pepT2* studies survived. Interestingly, all of our mutants displayed a decrease in bacterial fitness, being outcompeted by the wild-type strain in each case (**Figures 6A-C**).

For the *pepT1* mutant, we noted significantly diminished mutant cell accumulation in all organs. The most notable of these was in the brain where the *pepT1* mutant comprised only 8.3% of the total bacterial population (**Supplemental figure S3A**). Similarly, in the heart, we observed 13.5% of the total bacterial population resulting from the mutant strain (**Supplemental figure S3B**). We also saw significantly reduced survival of the mutant in the lungs (14.6%), kidneys (23%), spleen (21.6%) and liver (25.7%) (**Supplemental figures S3C-E**). Notably, we were unable to recover the wild-type or *pepT1* mutant cells from 5 brains, 1 spleen, and 2 livers. Furthermore, 2 hearts were solely colonized by the wild-type strain (**Figure 6A**).

For the *pepT2* mutant, we also noted significantly diminished mutant cell accumulation in all organs. The most prominent of which was the brain, where only 14.4% of the total bacterial population resulted from the mutant strain (**Supplemental figure S4A**). In the heart, we observed only 14.5% of the total bacterial burden consisting of mutant cells (**Supplemental figure S4B**). We also saw significantly reduced survival of the mutant in the lungs (16.5%), kidneys (18.6%), spleen (21%) and liver (23.1%) (**Supplemental figures S4C-F**).

When determining bacterial loads in the context of the *pepT1/pepT2* double mutant, we observed a profound reduction in the ability of this strain to survive within the host. Here there was significantly diminished mutant cell accumulation in all organs, most notably in the brain, where we observed 4.5% of the total bacterial burden consisting of mutant cells (**Supplemental figure S5A**). In the heart, we observed 5% of the total bacterial population resulting from the mutant strain (**Supplemental figure S5B**). We also saw significantly reduced survival of the mutant in the lungs (6.2%), kidneys (9.8%), spleen (5.9%), and liver (8.2%) and (**Supplemental figures S5C-F**). Notably in these studies, 3 brains, 4 hearts, 4 lungs, and 2 spleens were only colonized by the wild-type strain (**Figure 6C**).

## Discussion

In this study we identified two intracellular aminopeptidases from the M20B family as being important for full virulence in *S. aureus*. Aminopeptidases are ubiquitous in nature, cleaving proteins and peptides to liberate free amino acids; influencing processes ranging from aging and tissue repair to carcinogenesis and HIV infectivity (Taylor, 1993; Gonzales and Robert-Baudouy, 1996; Sapio and Fricker, 2014). In prokaryotes, aminopeptidases are best studied in lactic acid bacteria, where their ability to hydrolyze N-terminal amino acids facilitates growth on peptide-based substrates (Goldberg and Dice, 1974; Goldberg and St. John, 1976). Beyond a role in nutrition, recent work implicates aminopeptidases as key factors in virulence for a number of bacterial pathogens. For example, deletion of the secreted dipeptidyl aminopeptidase IV of *Porphyromonas gingivalis* attenuates virulence in murine models of infection (Kumagai et al., 2000). Similarly, deletion of Aminopeptidase N, PepN, from *Streptococcus pneumoniae* also attenuates virulence, whilst the purified protein itself elicits a strong host immune response (Kumagai et al., 2000; Wang et al., 2020). Interestingly, PepN has no signal sequence, however it is found in the secreted fractions of *S. pneumoniae* strains (Wang et al., 2020). Although not yet specifically determined, it would appear that these two enzymes mediate their role in disease by direct action on host proteins, given their extracellular nature. As with the PepT enzymes of *S. aureus*, however, examples exist of intracellular bacterial aminopeptidases also influencing virulence. These includes The M24 family PepP in *Pseudomonas aeruginosa* (Feinbaum et al., 2012), the M29 aminopeptidase Lmo1603 in *Listeria monocytogenes* (Cheng et al., 2015), and the M17 leucine aminopeptidase, LAP, from *S. aureus* (Carroll et al., 2012; Carroll Ronan et al., 2013). Interestingly, LAP and Lmo1603 are not required for growth in rich laboratory medium or those with peptides as the major nutritional source (as seen with the M20B family enzymes herein). Thus, it is thought that these enzymes influence virulence by the targeted bioactivation or inactivation of key signaling proteins within the cell that influence cellular homeostasis and/or virulence.

PepT enzymes were first isolated from *E. coli* and *Salmonella typhimurium* (Simmonds et al., 1976; Strauch and Miller, 1983), where they were shown to act only on tripeptides. From a specificity perspective, they will remove the N-terminal amino acid from most tripeptides except those containing an imino linkage across the target scissile bond. A structure exists for the *S. typhimurium* (PepT_ST_) enzyme, which is specifically classified by the Merops database (Rawlings et al., 2018) as an M20.003 type peptidase. Of the two *S. aureus* examples, PepT1 is also considered an M20.003 enzyme, whilst PepT2 is an M20.018 peptidase. When performing an alignment between PepT_ST_ and the two *S. aureus* enzymes, it is clear that all of the catalytic residues, as well as those required for Zn ion coordination, are shared by the three proteases (**Supplemental figure S6**). It is also clear that PepT1 shows the greatest homology with PepT_ST_. To explore the similarity of these enzymes further we performed predicted structure analysis of PepT1 and compared it to the published structure of PepT_ST_ (PDB ID: 1FNO) (Hakansson and Miller, 2002) (**Supplemental figure S7**). Here we note that the overall structures for PepT1 and PepT_ST_ are highly similar, including the catalytic pocket for both enzymes. The two PepT1 catalytic residues are located within an inner N-terminal pocket of the aminopeptidase. For the *S. aureus* variant, each of the catalytic residues, D80 and E173, are located within variable regions between the third and fourth β-sheets and between the sixth β-sheet and fifth α-helix, respectively. Similarly, the PepT_ST_ catalytic pocket is also located inside a N-terminal pocket, with the first catalytic residue also found within a variable region between the third and fourth β-sheets. However, the PepT_ST_ second catalytic residue, E172, is located within a small α-helix that is found between the eighth and sixth β-sheet and α-helix, respectively. Despite this, our structural analysis still indicates that the catalytic residues for both aminopeptidases mirror each other to form the catalytic pocket within the N-terminal domain. Thus, given the strong structural homology between these two enzymes, it would seem reasonable to suggest that PepT1 in *S. aureus* also likely functions as a tripeptidase.

Unlike PepT1, PepT2 is a M20.018 family enzyme, and is predicted to be a peptidase T-like protein. Searching within the MEROPS database, we were able to identify another metalloaminopeptidase within this same subclass, PepT from *Bacillus cereus* (PepT_BC_). While there is no current enzymatic information on the PepT_BC_ (or any other M20.018), the structure of this aminopeptidase has been deposited into the Protein Data Bank (PDB ID: 3GB0). Notably, the structure for the *S. aureus* PepT2 has also been solved and deposited into the Protein Data Bank (PDB ID: 3RZA). Therefore, we again performed a comparison analysis of these two proteins and found that the structures for PepT2 and PepT_BC_ are highly conserved, including each aminopeptidase’s catalytic pocket (**Supplemental figure S8**). The catalytic residues for each enzyme are located within a variable region in-between the third and fourth β-sheets (D81 for PepT2 and D79 for PepT_BC_) and within an α-helix that is in-between the sixth β-sheet and sixth α-helix (E143 for PepT2 and E170 for PepT_BC_). As with PepT1 and PepT_ST_, the catalytic residues for each aminopeptidase are found within a N-terminal catalytic pocket.

Given that no biochemical information exists on the M20.018 subclass, it is not clear if these proteins actually function as tripeptidase enzymes. Thus, although PepT1 appears to contain all the characteristics of a tripeptidase enzyme, it is not clear if PepT2 functions in this regard or if it in fact has a broader substrate preference as found for the wider M20 family (Lindner et al., 2003). We next compared the predicted structures of both PepT1 to the known structure of PepT2 from *S. aureus*. Here we found that while the overall structure is conserved, PepT1 harbors an additional α-helix that is found between the fourth and fifth β-sheets within this aminopeptidase and is located on the outside of the N-terminal catalytic pocket (amino acid residues 118-123) (**Supplemental figure S9**). Therefore, based upon the structural dissimilarity of the PepT1 and PepT2 catalytic pockets, this would suggest that each aminopeptidase is able to recognize and interact with its own diverse set of substrates. This suggests that both enzymes may function independently from each other in terms of substrate recognition and catalysis. This notion is supported by the finding that only PepT2 can be detected in the cell membrane fraction (albeit at lower levels than in the cytosol), despite having no transmembrane domains.

To test this notion, we compared the viability of each mutant from different niches within our co-infection models (**Supplemental figure S10**). Interestingly, in the brain, the percent recovery of the *pepT1* mutant strain was nearly half that for the *pepT2* mutant. This suggests that the PepT1 protease is perhaps the more important of the two enzymes in this organ, and may account for the diminished survivability of the double mutant. For the heart, lungs, kidneys, spleen, and liver the single mutants have very similar percent survival, however the double mutant recovery is significantly lower than merely half that of the single mutant strains. This suggests that each enzyme is contributing its own, non-overlapping function in these niches; the loss of which results in a major reduction in the ability of *S. aureus* to survive and/or disseminate.

Recent work surrounding co-infection models has introduced the concept of population bottlenecks of infection in which only a few ‘founder cells’ break through various immune defenses, leading to increased clonality within organs and possible bias in resulting bacterial burden data. These bottlenecks have been reported to primarily stem from very few cells surviving an immune challenge from liver Kupffer cells, allowing any surviving cells to then colonize the kidney (Prajsnar et al., 2012; Pollitt et al., 2018). However, while it is thought that one bacterial strain will out compete the other after co-infection based on chance, numerous studies have shown consistencies in virulence phenotypes from models of co-infections (Vimont et al., 2012; Weiss et al., 2014; Misawa et al., 2015; Copin et al., 2019; Lonergan et al., 2019). While the information gleaned from bottleneck studies has provided insightful regarding the spatial and temporal progression of *S. aureus* infection within the host, these breakpoints do not eliminate overall differences in pathogenicity between strains when sufficient inocula are utilized (Weiss et al., 2014; Misawa et al., 2015; Copin et al., 2019). This is observed across species and infection types, solidifying its reliability in myriad *in vivo* conditions (Auerbuch et al., 2001; Winstanley et al., 2009; Hood et al., 2012; Linkevicius et al., 2016; Young et al., 2020). Furthermore, in every test used herein – be it *ex vivo* or *in vivo* – the *pepT* mutants are always impaired in their ability to cause disease compared to the parental strain; something which random chance alone would not produce. Therefore, the murine co-infection model of systemic infection utilized in this study directly reflects the attenuation in virulence observed, ex vivo, for our *pepT* mutants.

**Figure 7:**
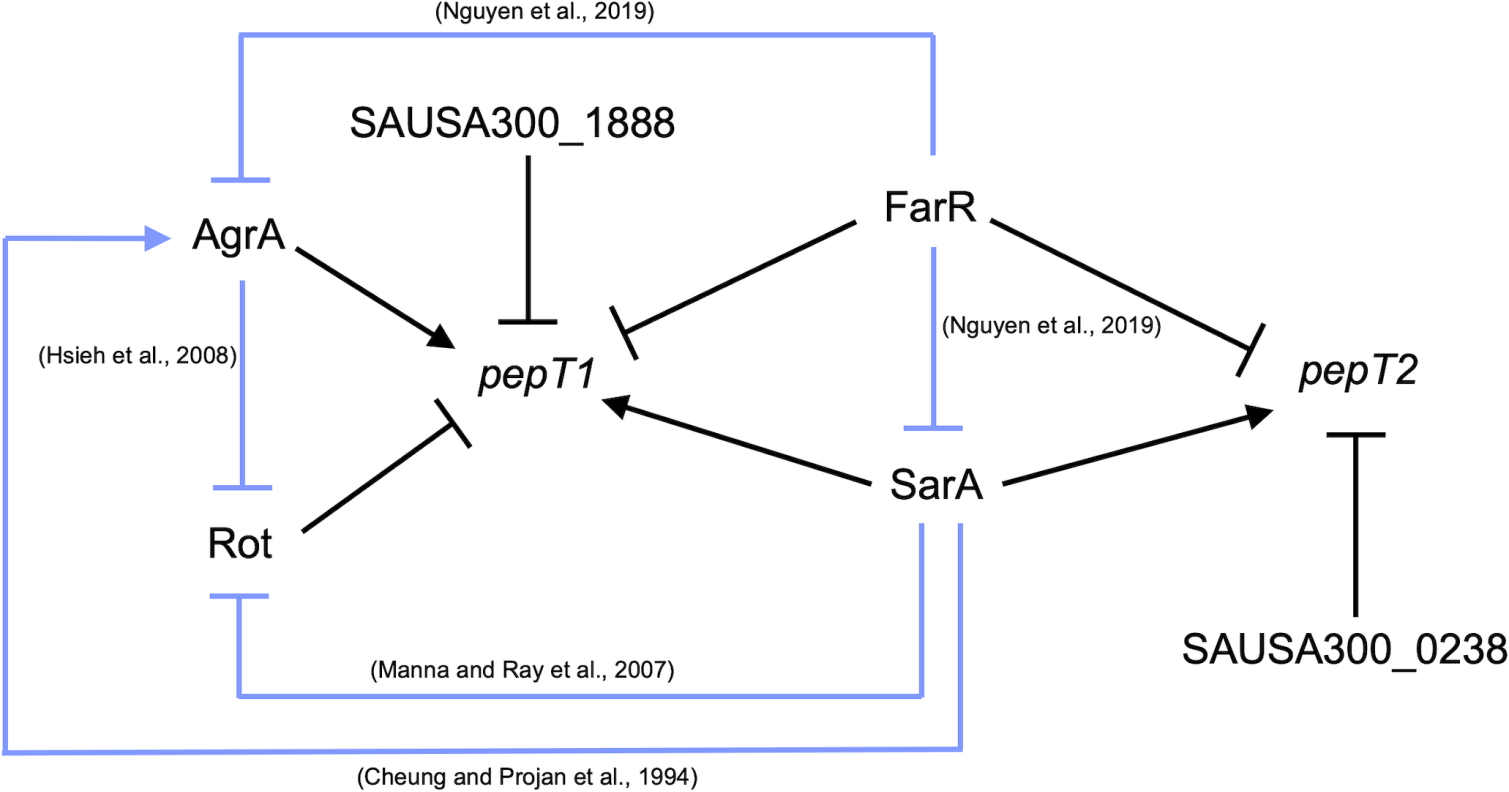
*pepT1* and *pepT2* expression is controlled by known regulators of virulence. Regulatory map for *pepT1* and *pepT2* transcriptional regulation. Black lines denote new regulatory pathways identified in this study. Light blue lines represent regulatory pathways identified in previous works with references.

When considering the expression and regulation of *pepT1* and *pepT2*, we note that both aminopeptidases are positively influenced by SarA, with *pepT1* expression also being activated by AgrA. Interestingly, we found that FarR, the regulator of fatty acid resistance, negatively affects *pepT1* and *pepT2* expression. FarR is part of the TetR family of regulators and was shown to repress both *sarA* and *agr*, with the inactivation of *farR* resulting in increased hemolysis and pathogenicity in a murine pneumonia model (Nguyen et al., 2019). Thus, in the absence of FarR, both SarA and AgrA would be de-repressed. This would then allow for SarA to activate AgrA (Cheung and Projan, 1994) resulting in activation of *pepT1* and *pepT2* expression (**Figure 7**). In addition, we found that Rot, the repressor of toxins (Mcnamara et al., 2000), also negatively affects *pepT1* expression. Notably, both SarA and AgrA have been shown to repress Rot transcription and translation, respectively (Manna and Ray, 2007; Hsieh et al., 2008). Therefore, once either AgrA or SarA is activated, this would in turn repress Rot, resulting in the de-repression of *pepT1*. While further investigation is needed to determine if the repression of *pepT1* and *pepT2* by FarR is the start of the regulatory cascade that dictates their activation, it is clear that major mediators of virulence factor expression also oversee production of these important aminopeptidases.

In summary, we have identified two M20B aminopeptidases that are integral to *S. aureus* pathogenesis, as their loss results in the severe attenuation in virulence for both *ex vivo* and *in vivo* models of infection. However, unlike many intracellular aminopeptidases, neither PepT1 nor PepT2 are essential for nutrient acquisition in peptide rich media. While the exact role of PepT1 and PepT2 remains to be elucidated, our data supports the necessity for identifying the protein and/or peptide targets that each of these proteases cleave in order to understand their important virulence impacting functions.

## Data availability statement

The original contributions presented in the study are included in the article/**Supplementary Material**. Further inquiries can be directed to the corresponding author.

## Ethics statement

All animal studies was performed under the approval of the University of South Florida's Institutional Animal Care and Use Committee by trained lab personnel.

## Author contributions

DR and LS conceived the original study. NT, DR, MR and JJ performed experiments. BT performed molecular modeling. WY provided reagents. NT, DR and M-EJ wrote the original draft. LS edited the manuscript and provided funding. All authors contributed to the article and approved the submitted version.
