# Supplementary material for: The identification of two M20B family peptidases required for full virulence in Staphylococcus aureus
Source: Front Cell Infect Microbiol. 2023 Jul 19;13:1176769. doi: 10.3389/fcimb.2023.1176769 (PMC10394242; doi:10.3389/fcimb.2023.1176769)
Supplement: Supplementary file 1 [file DataSheet_1.pdf]

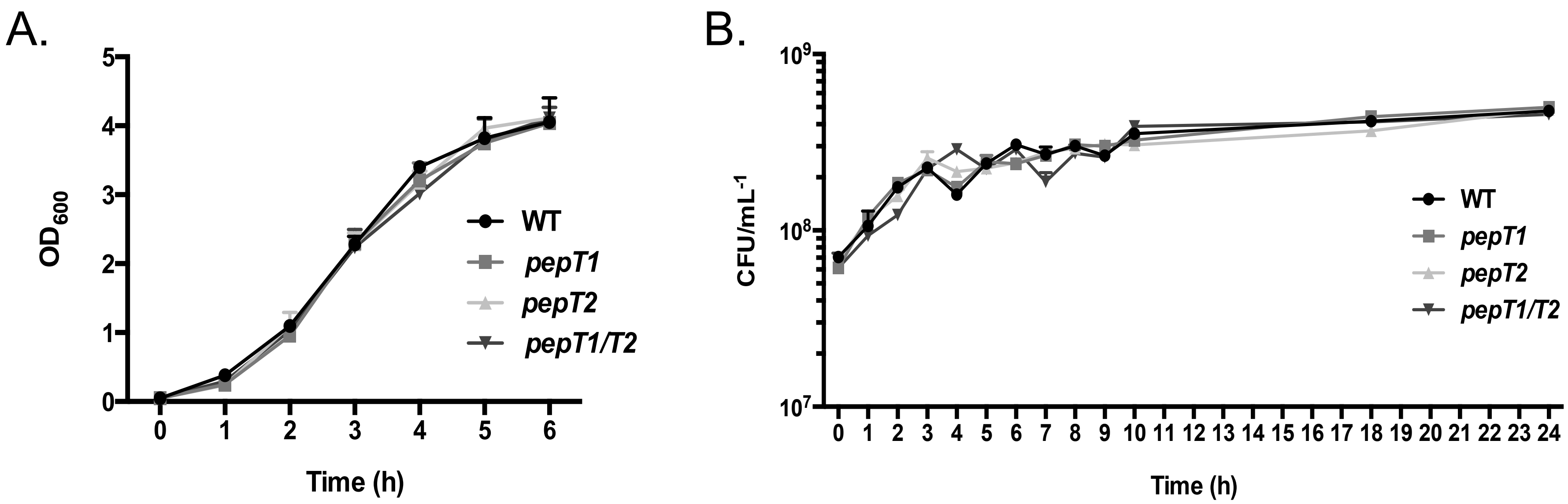

Supplemental Figure S1: *S. aureus* PepT Enzymes are not Required for Growth in TSB or Peptide Rich Media. (A) Exponentially growing wild-type, *pepT1, pepT2,* and *pepT1/T2* double mutant strains were separately inoculated, in triplicate, into TSB at a starting OD_{600} = 0.05. Strains were grown at 37°C with shaking and samples were taken every hour to determine OD_{600} values. (B) As in A, but strains were inoculated into 10% skim milk. Samples were taken every hour, and cell viability assessed by calculating CFU/mL. Error bars are shown ±SEM.

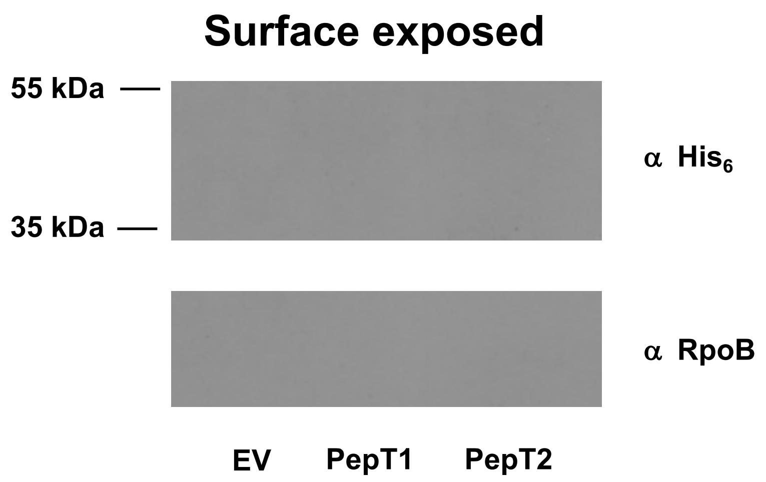

Supplemental Figure S2: *S. aureus* PepT Enzymes are not located on the cell surface. Immunoblots using an anti-His antibody were used to determine if either PepT1 or PepT2 were located on the cell surface. The surface exposed cell fraction was collected after 15h of growth from *pepT1* and *pepT2* mutants expressing the relevant variant of PepT1 enzyme bearing a His_6 tag. Immediately following growth, all strains were adjusted to equal optical densities prior to cell fractionation. An antibody specific to the RNA polymerase B subunt (RpoB) was used as a cytoplasmic control. Wild-type USA300 HOU harboring an empty pMK4 vector (EV) was also used as a control.

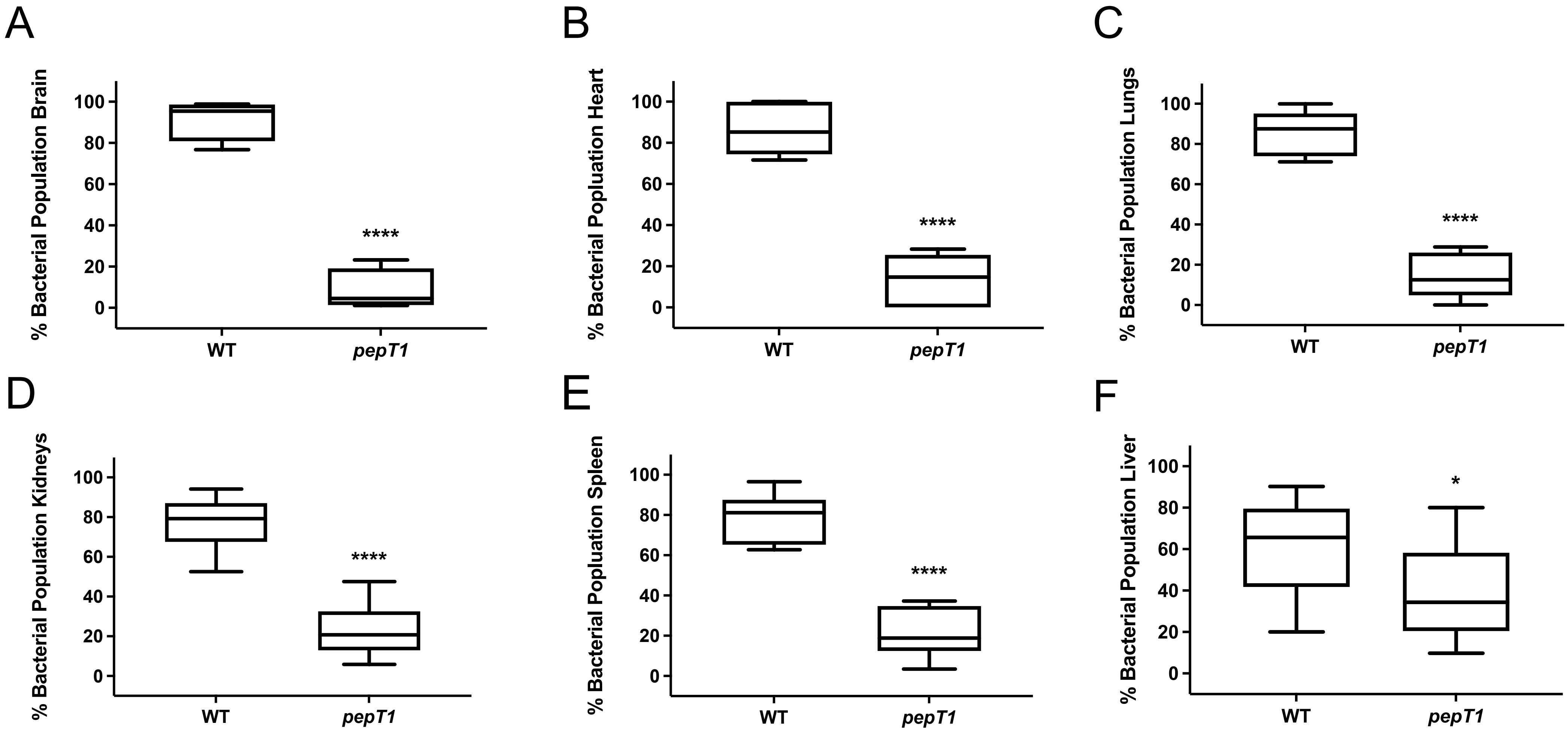

Supplemental figure S3: The *S. aureus pepT1* enzyme is required for disease causation in a murine model of septic infection. Mice were co-infected with 5 x 10^7 CFU/mL of the wild-type and *pepT1* mutant strains in a 1:1 ratio. The infection was allowed to progress for 7 days and the mice were subsequently euthanized. One mouse died before the conclusion of the infection period and was excluded from the presented data. The percent of the bacterial population was quantified for the (A) brain, (B) heart, (C) lungs, (D) kidneys, (E) spleen, and (F) liver. The data is represented as box-and-whisker plots using median values represented as a black line in the center of the box. A student's t-test with Welch's correction was used to assess statistical significance in comparison to the wild-type, (*, p < 0,05; ****, p < 0.0001). All data is derived from 9 mice. Error bars are shown ± SEM. Organs that did not contain any bacterial loads are not represented.

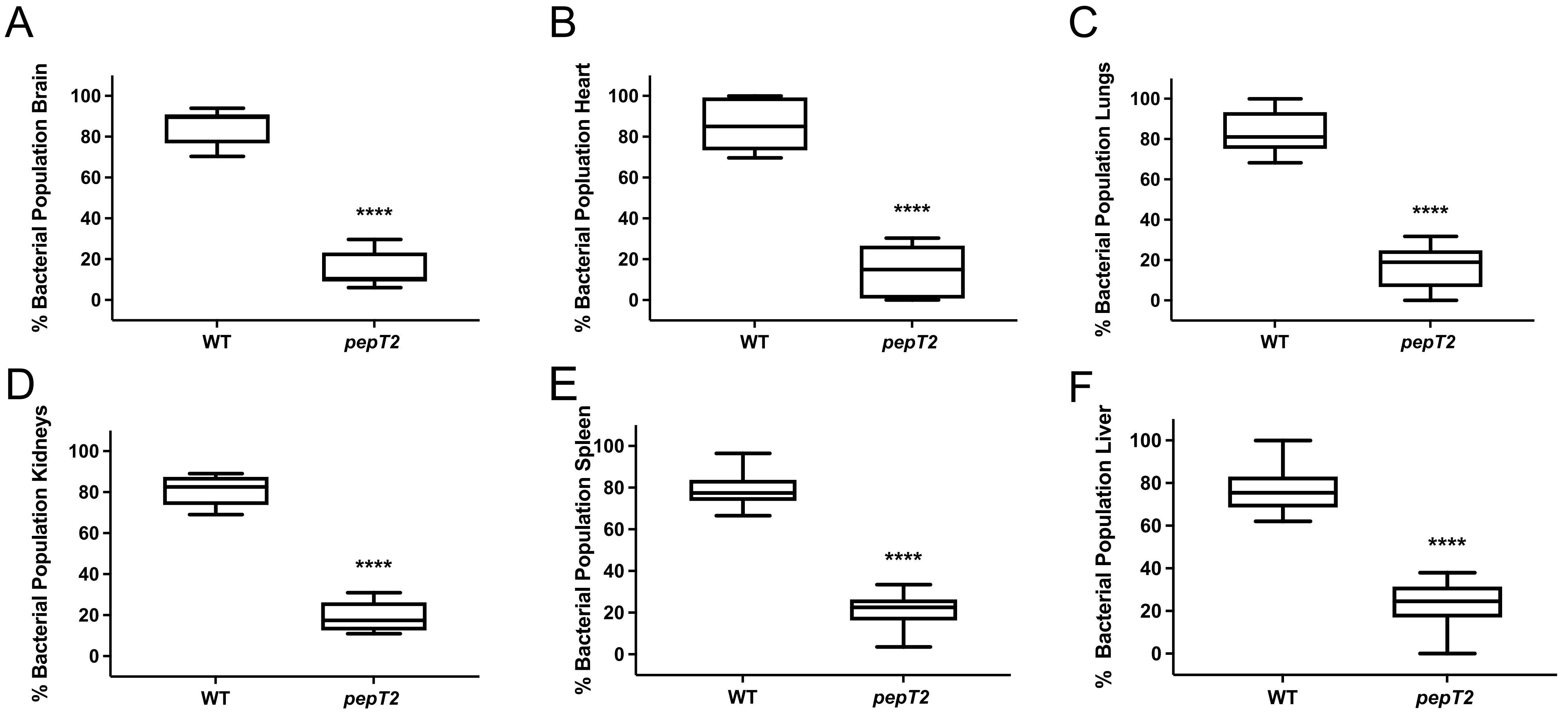

Supplemental figure S4: The *S. aureus pepT2* enzyme is required for disease causation in a murine model of septic infection. Mice were co-infected with 5 x 10^7 CFU/mL of the wild-type and *pepT1* mutant strains in a 1:1 ratio. The infection was allowed to progress for 7 days and the mice were subsequently euthanized. The percent of the bacterial population was quantified for the (A) brain, (B) heart, (C) lungs, (D) kidneys, (E) spleen, and (F) liver. The data is represented as box-and-whisker plots using median values represented as a black line in the center of the box. A student's t-test with Welch's correction was used to assess statistical significance in comparison to the wild-type, (****, $p < 0.0001$). All data is derived from 10 mice. Error bars are shown ± SEM. Organs that did not contain any bacterial loads are not represented.

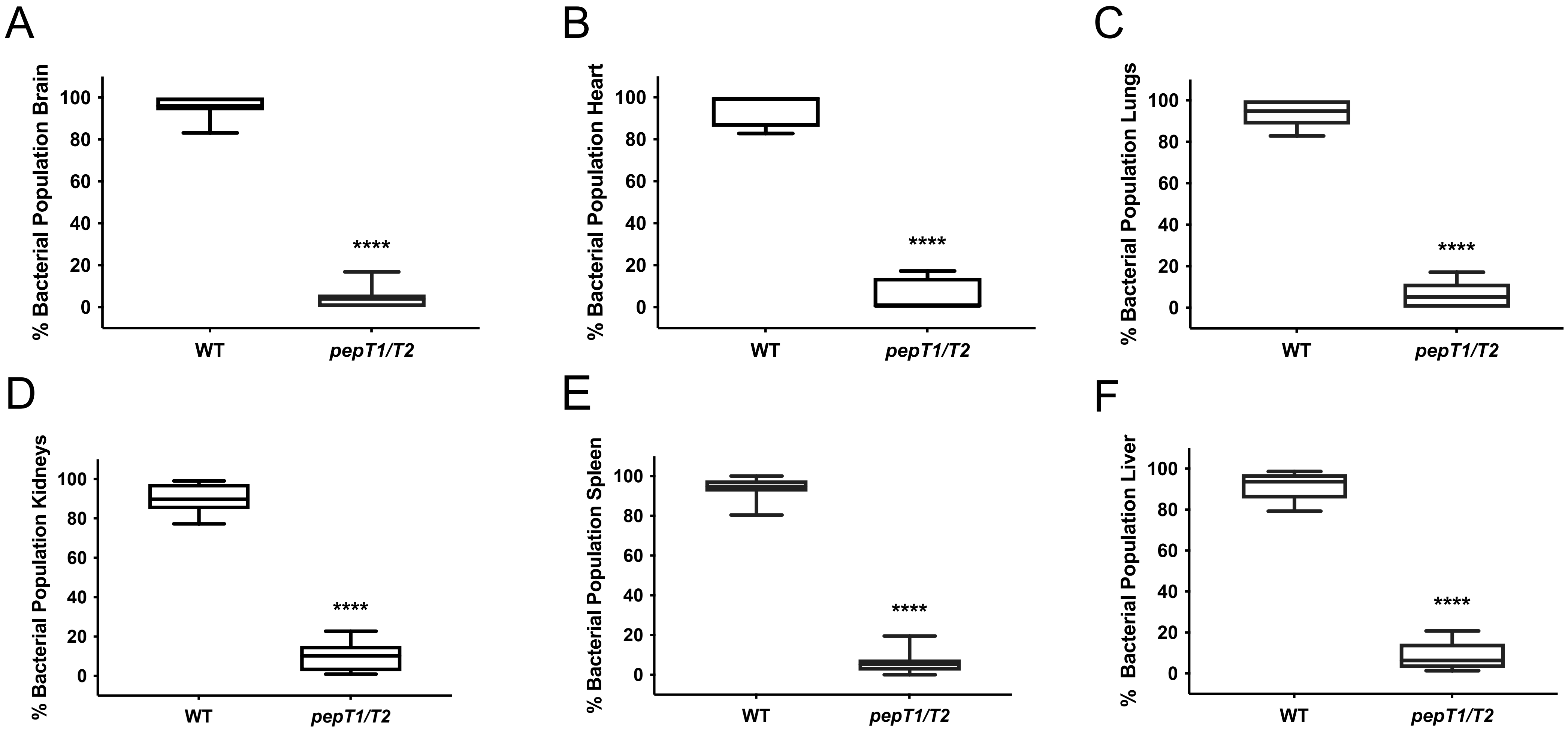

Supplemental figure S5: The *S. aureus* PepT enzymes are required for disease causation in a murine model of septic infection. Mice were co-infected with 5 x 10^7 CFU/mL of the wild-type and *pepT1* mutant strains in a 1:1 ratio. The infection was allowed to progress for 7 days and the mice were subsequently euthanized. The percent of the bacterial population was quantified for the (A) brain, (B) heart, (C) lungs, (D) kidneys, (E) spleen, and (F) liver. The data is represented as box-and-whisker plots using median values represented as a black line in the center of the box. A student's t-test with Welch's correction was used to assess statistical significance in comparison to the wild-type, (****, $p < 0.0001$). All data is derived from 10 mice. Error bars are shown ± SEM. Organs that did not contain any bacterial loads are not represented.

```
PepT2      MINEQRLLNTFFELVQIDSETGNESTIQPILKEKFIALGLDVKEDEAAKHP--KLGANN-    57
PepT       ---MDKLLERFLHYVSLDTQSKSGVRQVPSTEGQWKLLRLLKQQLEEMGLVNITLSEKGT    57
PepT1      --MKNQLIDRLTRYTTIDTQSDPKSTTTPSTEKQWDLLHLLEKELQQLGLP-TDLDENGY    57
               ::*:: : . . :*:::       *  : ::  * *  :: :        *. :.

PepT2      LVCTMNSTIEEGEVPKLYLTSHMDTVV--PAINVKP------------------------    91
PepT       LMATLP-ANVEGDIPAIGFISHVDTSPDFSGKNVNPQIVENYRGGDIALGIGDEVLSPVM    116
PepT1      LFATLE-SNIDVDVPTVGFLAHVDTSPDFNASNVKPQIIENYDGKPYKLGNTKRVLDPKV    116
           *..*:  :  : ::* : : :*:**     . **:*

PepT2      -----IVKDDGYIYSDGTTILGADDKAGLAAMLEVLQVIKEQQ-IPHGQIQFVITVGEES    145
PepT       FPVLHQLLGQTLITTDGKTLLGADDKAGVAEIMTALAVLKGNP-IPHGDIKVAFTPDEEV    175
PepT1      FPELNSLVGHTLMVTDGTSLLGADDKAGIVEIMEAICYLQEHPEIKHGTIRIGFTPDEEI    176
                 : ..  : :**.::********:. :: .:  :: :  * ** *:. :* .**

PepT2      GLIGAKELNSELLDADFGYAIDASADVGTTVVGAPTQMLISAKIFGKTAHASTPKEG-VS    204
PepT       G-KGAKHFDVEAFGAQWAYTVDGGGVGE-LEFENFNAASVNIKIVGNNVHPGTAKGVMVN    233
PepT1      G-RGPHKFDVDRFNADFAYTMDGSQYGE-LQYESFNAAEAVITCHGVNVHPGSAKNAMVN    234
           *  * :.:: : :.*::.*::*..           .      .  * ..* .: *   *.

PepT2      AINIAAKAISRMKLGQVDEITTANIGKFHGGSATNIVADEVILEAEARSHDPERIKTQVK    264
PepT       ALSLAARIHAEVPADEAPETTEGYEGFYHLASMKGTV-DRAEMHYIIRDFDRKQFEARKR    292
PepT1      AIRLGEQFDSLLPDSEVPERTEGYEGFYHLMNFEGTV-EKATLQYIIRDHDKKQFELRKK    293
           *: :. :  : :  .:. * * .  * :*  .  . * :.. :.   *..* :::: : :

PepT2      HMTDVFETTASELGGKAVV--TVEQSYPG--FKINDNEAVVKIAQESARNLGLSANTIIS    320
PepT       KMMEIAKKVGKGLHPDCYIELVIEDSYYNMREKVVEHPHILDIAQQAMRDCHITPEMKPI    352
PepT1      RILEIRDDINAHF-ENYPVKVDISDQYFNMAEKILPLPHIIDIPKRVFAKLDIPANTEPI    352
           :: :: .     :  .  :   :.:.* .   *:     ::.* :.   .  :  :

PepT2      GGGSDGSIINTFGIPSVILGVGYEKIHTTNERMPIKSLNLLASQVLEIIKIVARHSK    377
PepT       RGGTDGAQLSFMGLPCPNLFTGGYNYHGKHEFVTLEGMEKAVQVIVRIAELTAKRGQ    409
PepT1      RGGTDGSQLSFMGLPTPNIFTGCGNFHGPYEYASIDVMEKAVQVIIGIVEDIAENH-    408
            **:**: :. :*:*   : .*  : *   *   :. ::  ..  :: * :  *..
```

Supplemental Figure S6: Amino acid sequence alignment for PepT1, PepT2 and PepT from *S. typhimurium*. Amino acid sequences for PepT1, PepT2, and PepT from *S. typhimurium* were aligned using Clustal omega. Fully conserved residues are indicated by (*). Residues with a score > 0.5 or with a score ≤ 0.5 on the PAM 250 matrix are denoted by a (:) and (.), respectively. Blue and yellow highlighted residues denote catalytic amino acid residues and Zn binding sites, respectively.

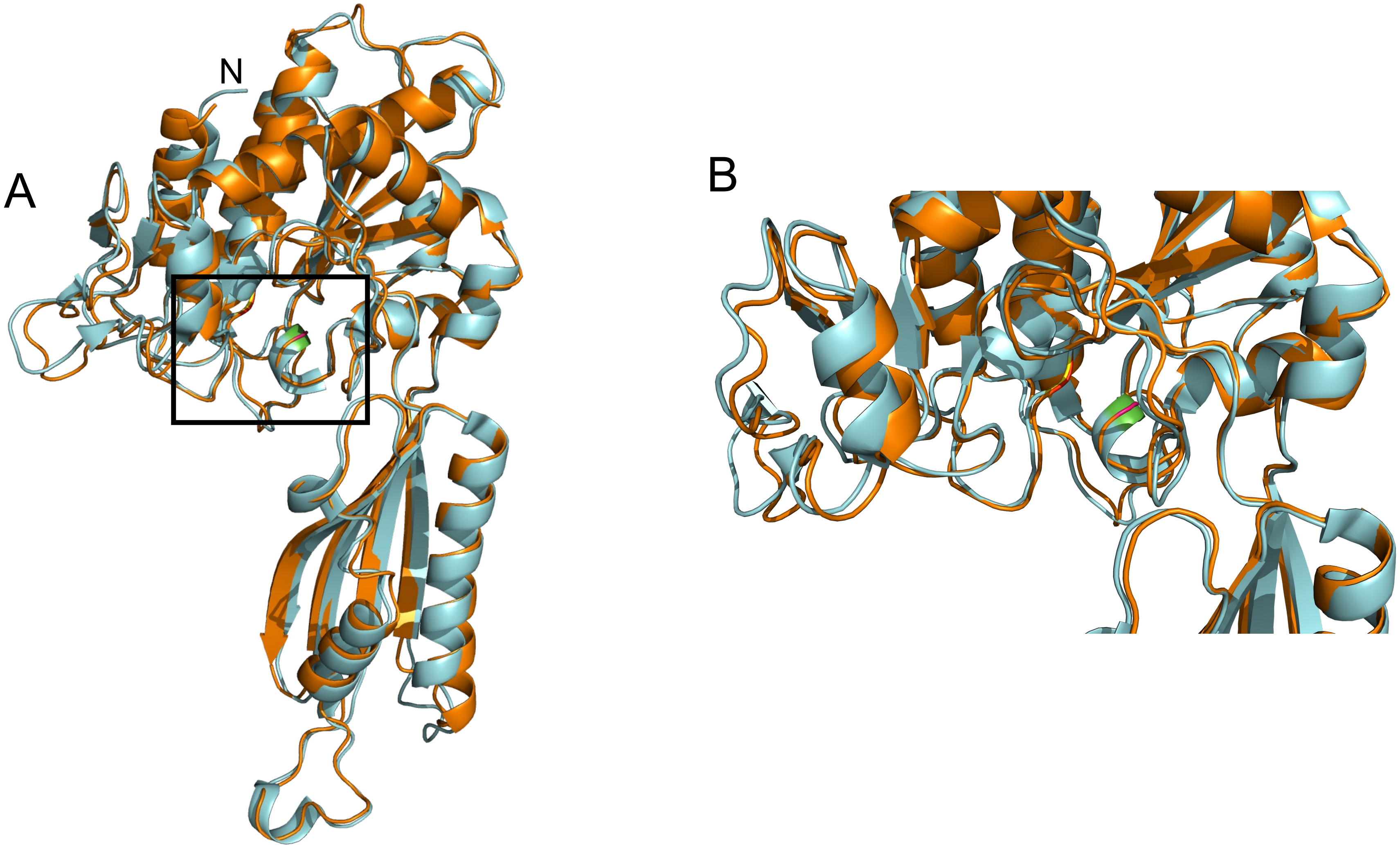

Supplemental Figure S7: *S. aureus* PepT1 is structurally similar to *S. typhimurium* PepT. (A) The predicted structure for PepT1 from *S. aureus* derived from Alphafold (orange) was overlayed with the solved structure of PepT from *S. typhimurium* (light blue) (Uniprot ID: 1fno) using PyMol (version 2.0). The black box denotes the catalytic pocket for both aminopeptidases. N denotes the start of the N-terminus for each enzyme. (B) Close up image of the catalytic pocket for both PepT1 from *S. aureus* (orange) and PepT from *S. typhimurium* (light blue). Red (D80) and magenta (E173) residues denote catalytic sites for PepT1 from *S. aureus*. Yellow (D80) and green (E172) residues denote catalytic sites for PepT from *S. typhimurium*.

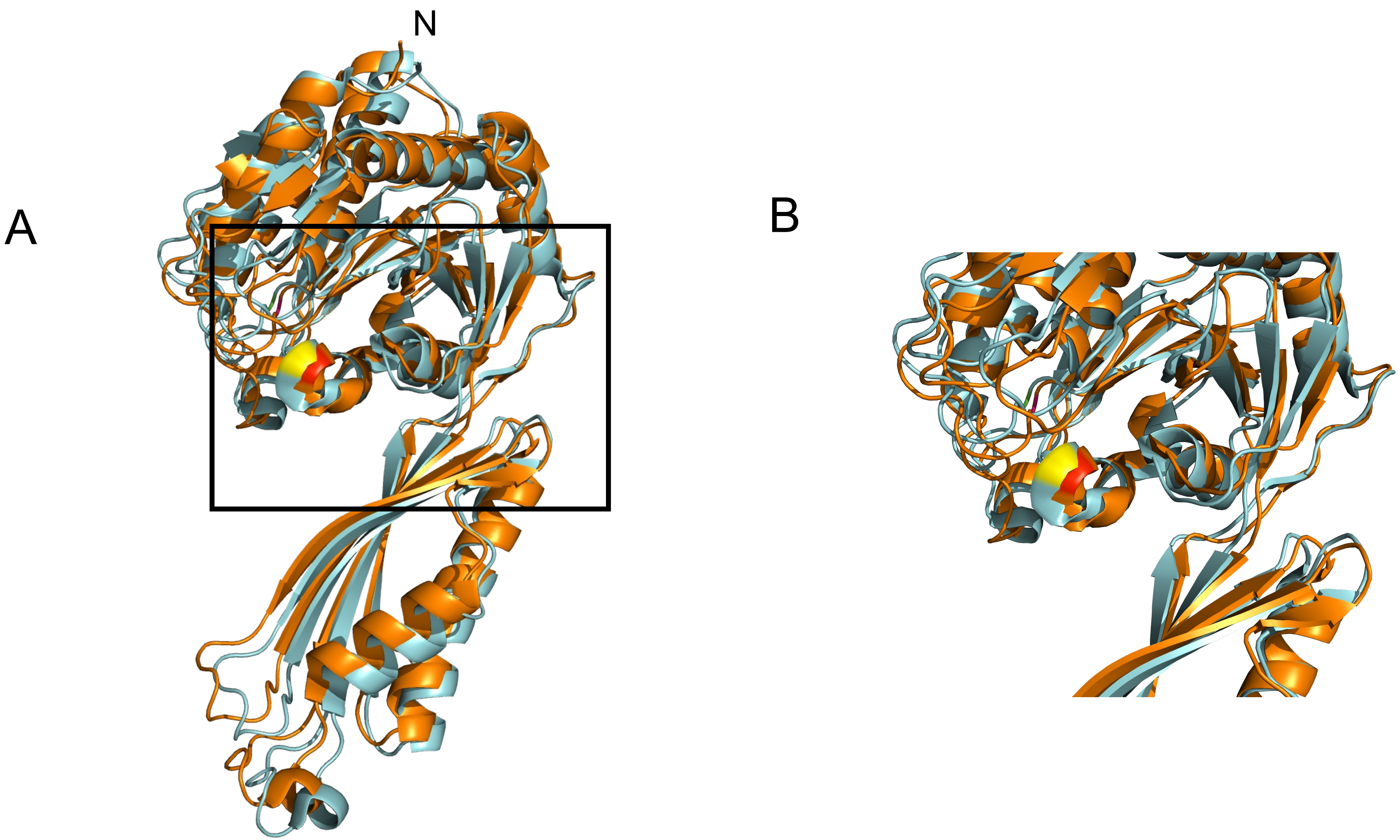

Supplemental Figure S8: *S. aureus* PepT2 is structurally similar to *B. cereus* PepT. (A) The solved structure for PepT2 from *S. aureus* (PDB: 3RZA) (orange) was overlayed with the solved structure of PepT from *B. cereus* (light blue) (PDB ID: 3GB0) using PyMol (version 2.0). The black box denotes the catalytic pocket for both aminopeptidases. N denotes the start of the N-terminus for each enzyme. (B) Close up image of the catalytic pocket for both PepT2 from *S. aureus* (orange) and PepT from *B. cereus* (light blue). Magenta (D81) and red (E143) residues denote catalytic sites for PepT2 from *S. aureus*. Green (D79) and yellow (E140) residues denotes catalytic sites for PepT from *B. cereus.*

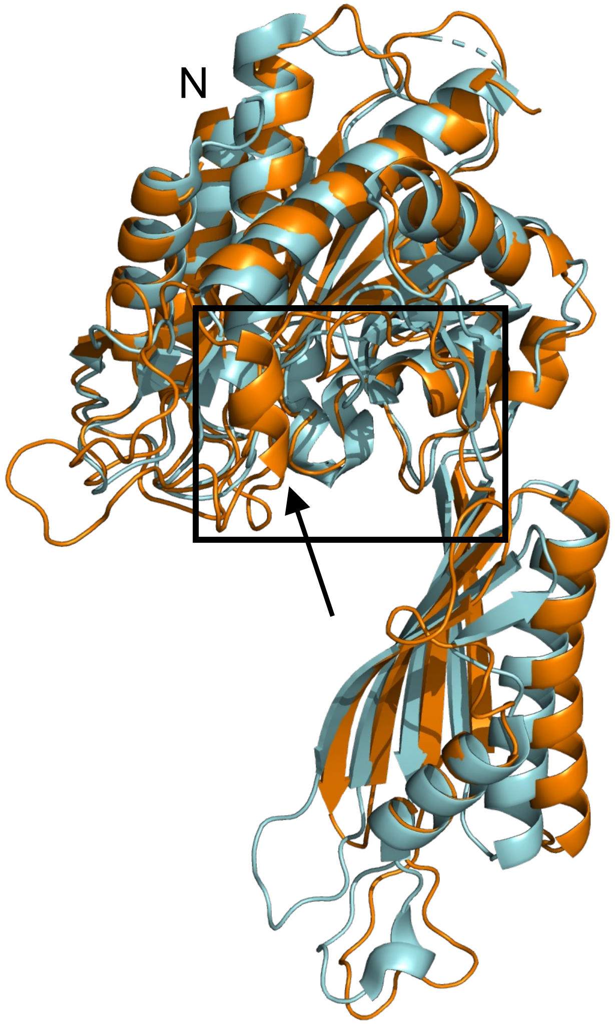

Supplemental Figure S9: ***S. aureus*** **PepT1 and PepT2 have different substrate binding pockets.** The predicted structure for PepT1 was generated using Alphafold (orange) and overlayed with the solved structure of PepT2 (light blue) (PDB: 3RZA) using PyMol (version 2.0). The black arrow denotes the predicted alpha helix (residues 118-123) in the catalytic pocket for PepT1 not present in the catalytic pocket for PepT2. The black box denotes the catalytic pocket for both aminopeptidases. N denotes the start of the N-terminus for each enzyme.

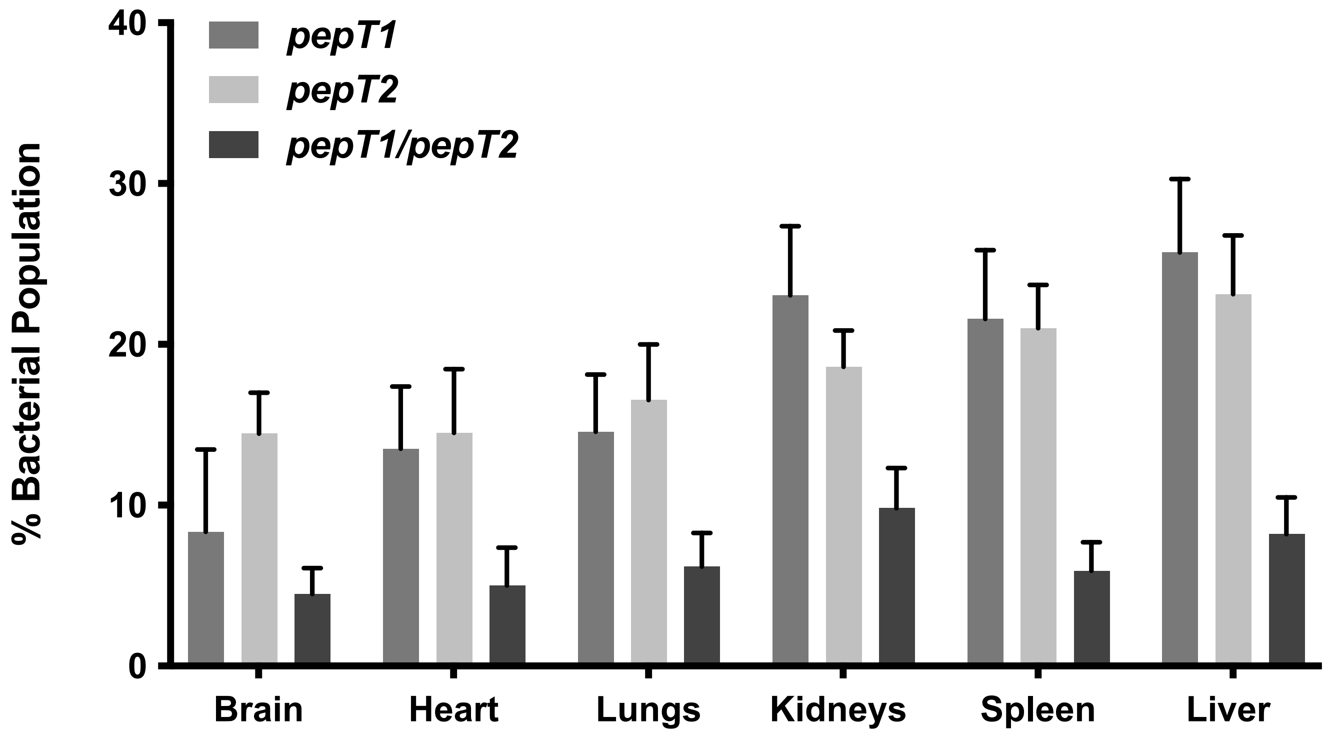

Supplemental Figure S10: Comparison of virulence for single and double *pepT* mutants. The percent of the bacterial population of the *pepT1*, *pepT2*, and *pepT1*/*T2* double mutants inside the brain, heart, lungs, kidneys, spleen, and liver. Data is from Supplemental Figures S3-5.
